# Latent bloodstain detection using a selective turn-on NIR fluorescence dye responsive to serum albumin†

**DOI:** 10.1039/d3ra04320g

**Published:** 2023-09-15

**Authors:** Jing Qu, William Meador, Pohlee Cheah, Eden E. L. Tanner, Jared Delcamp, Yongfeng Zhao

**Affiliations:** a Department of Chemistry, Physics & Atmospheric Sciences, Jackson State University Jackson MS 39217 USA yongfeng.zhao@jsums.edu; b Department of Chemistry and Biochemistry, University of Mississippi, University MS 38677 USA delcamp@olemiss.edu

## Abstract

Bloodstain detection can provide crucial information and evidence at a crime scene; however, the ability to selectively detect bloodstains in a non-destructive manner with high sensitivity and low background is limited. This study reports a fluorescent dye (sulfonate indolizine squaraine, SO_3_SQ) for bloodstain visualization under near-infrared (NIR) irradiation. While the dye itself is minimally fluorescent in aqueous solution, it exhibits a “turn-on” mechanism upon binding with human serum albumin (HSA) as the fluorescence intensity increases over 160 times with strong absorption and emission at 693 nm and 758 nm, respectively. Bloodstains can be visualized on a surface even after being diluted 1000 times, and washed latent bloodstains can be detected with high sensitivity. Further, the turn-on fluorescent emission lasts for a minimum of seven days, allowing adequate time for detection. This study also indicates that the SO_3_SQ can sensitively detect bloodstain after the bloodstain aged for one week. Furthermore, the detection of latent blood fingerprint patterns from colorful backgrounds is demonstrated using this non-destructive method. The selective turn-on fluorescent dye with NIR excitation and emission is highly suitable in forensic science for bloodstain visualization.

## Abstract 2

Latent bloodstains can be visualized using a selective turn-on NIR fluorescence dye responsive to serum albumin. This non-destructive method can detect aged bloodstains and image latent blood fingerprint patterns against colorful backgrounds.

## Graphical abstract

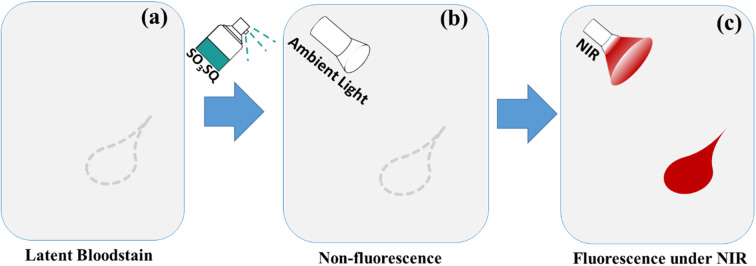

## 1. Introduction

Forensic analysis has successfully improved public security by systematically collecting trace evidence.^1,2^ One of the main areas of focus in forensic investigations are the bodily fluids obtained at a crime scene,^3,4^ including saliva,^5^ urine,^6^ blood,^7^ and sweat.^8^ Among fluid types, blood is the most commonly studied piece of biological evidence.^9^ The appearance of blood at a crime scene provides critical information for forensic practitioners,^10^ including the pattern of a blood splatter.^11^ Further, blood is a source of deoxyribonucleic acid (DNA), which can be used to identify a person of interest.^12–15^ In practice, blood is usually collected for DNA testing after being located by an imaging technique.

Because of the high demand for bloodstain detection, various methods have been developed for bloodstain including colorimetric and luminescent methods.^16–19^ Specifically, advanced photography techniques with alternate light sources (ALS) are used mainly as an aid to enhance the bloodstain for photographic purposes. If bloodstains cannot be found by ALS methods because of poor sensitivity, a colorimetric search is often undertaken. Chemicals such as 3,3′,5,5′-tetramethylbenzidine (TMB),^20^ leucomalachite green (LMG),^21^ and phenolphthalein (Kastle–Meyer)^22–24^ are commonly used for this technique. The principle is based on an observed color change after contact with blood. However, further development of this technique is impeded by the relatively low sensitivity and poor contrast.

To increase the sensitivity, detection strategies based on luminol and fluorescein have been employed as a major methodology for bloodstain detection. By taking advantage of the catalytic oxidation activity of hemoglobin in blood, luminol has high sensitivity towards bloodstain detection.^25^ The disadvantage of luminol is that the chemiluminescence must be observed in a dark environment and that the observation window is extremely short (on the order of seconds). Further, multiple applications of luminol degrade the bloodstain patterns. This makes the detection of bloodstains with luminol a highly impractical method. Fluorescein has been considered as an alternative to luminol because of a favorably high quantum yield of fluorescein and commercially available lasers matching its excitation wavelength. During the visualization process, the fluorescin (a fluorescein derivative) is oxidized to release fluorescein in the presence of hemoglobin in blood.^26^ The intensity of the signal can be further enhanced by hydrogen peroxide. Although the two methods mentioned are useful, they suffer from several limitations. The first limitation is that false positives with fluorescence derivatives can arise in the presence of other catalysts in the background, including metal ions (rust, soil, bacteria, or plant pigments) or oxidants (bleach, horseradish sauce, and some fruits). Second, both methods require the use of fresh reagents. For example, fluorescein derivative requires zinc activation to increase its sensitivity, and luminol needs storage at low temperatures and away from light due to inherent instability of the molecule. Lastly, both methods involve harsh conditions that damage DNA in the bloodstain.^27,28^ Recently, the fluorescence turn-on strategy under UV irradiation has been demonstrated to increase selectivity by designing fluorescent dyes that bind to human serum albumin (HSA), a protein which is abundant and specific to blood samples.^29^ The high resolution and reliability of the method is due to the formation of a covalent bond between tetraphenylethene maleimide (TPE-MI) and a free thiol group located in a binding pocket of human serum albumin (HSA).

Recently, near infrared (NIR) dyes have been reported to help overcome background issues on fingerprint visualization, demonstrating a great advantage over UV and visible region dyes by enhancing the sensitivity.^30^ Blood strongly absorbs UV light (300–400 nm) which causes background interference with UV fluorophores due to the inherent fluorescence of blood under UV irradiation.^31,32^ Blood does not absorb or emit appreciably in the NIR (700–1000 nm) though, resulting in a significant reduction in the background interference that is encountered with visible and UV light sources. The NIR fingerprint powder has been shown to facilitate complete background removal during fingerprint imaging.^33,34^ This NIR fluorescent powder has provided highly effective background interference removal and allows for the visualization fingerprints on substrates with challenging backgrounds (*i.e.* plastic banknotes).^35^ In addition, several studies have focused on comparison methods for analyzing the performance of NIR and UV emission spectroscopy for blood detection and found that NIR emission is more effective at detecting blood and is more specific to blood.^36^ Similarly, other studies have reported NIR excitation enhances image contrast in forensic investigations by eliminating background fluorescence.^37–39^ Therefore, bloodstain detection agents in the NIR region are promising technologies with little exploration in the literature.^30^

**Scheme 1:**
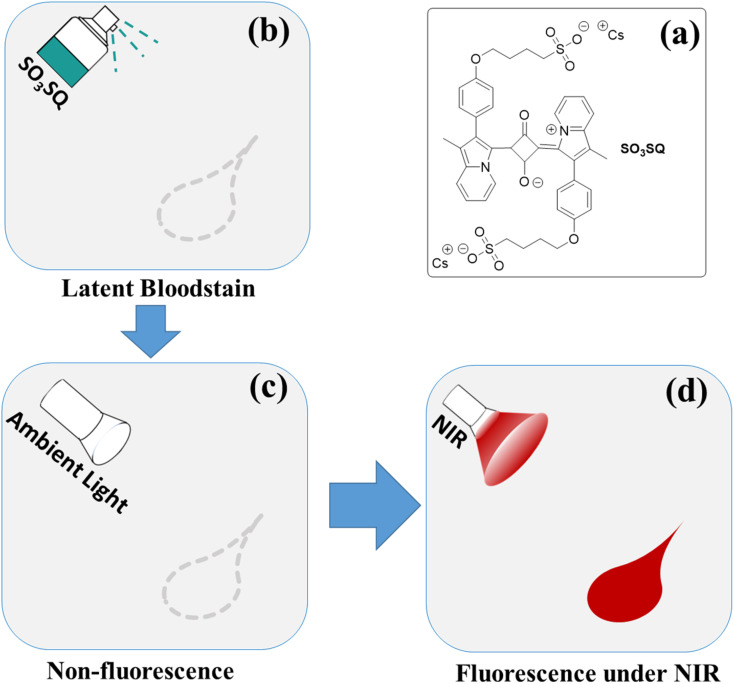
The principle for visualization of latent bloodstain: (a) the chemical structure of SO_3_SQ; (b) latent bloodstain pattern; (c) non-fluorescent SO_3_SQ under ambient light; (d) visualization of the bloodstain under NIR light.

Herein, this study reports on a novel strategy for bloodstain detection by combining the turn-on property of a fluorescent dye with its absorption and emission in the NIR region. The detection process is based on the interaction between albumin and our recently reported SO_3_SQ.^40^ SO_3_SQ is relatively non-emissive under ambient light, however, strong fluorescence emission can be obtained when interacting with blood under NIR irradiation (Scheme 1). Using this fluorescence turn on strategy, DNA is completely preserved. The enhancement in fluorescence of SO_3_SQ may derive from the disaggregation of dyes^41,42^ and subsequently binding of monomeric SO_3_SQ to albumin.^43^ The change in environment after complexation with the HSA protein might also promote the high fluorescent emission. To the best of our knowledge, there is no report on the latent bloodstain detection using NIR fluorescent dyes with turn-on fluorescent intensity enhancements.

## 2. Experimental section

### 2.1 Materials and characterization

Absorption spectra were taken using a UV-2600 spectrometer (Shimadzu, Japan), over a range of 200–1000 nm. The fluorescence and specificity properties of this method were studied using a fluoromax-4 fluorimeter (Horiba Scientific, USA). The bloodstain detection performance is evaluated using a 660 nm red light LED flash (Aukvi, China) to irradiate the samples. Digital pictures were taken by Kolari Pocket Full-Spectrum Converted Infrared Photography Camera with 3-Filter Starter Kit (Kolari Vision, New Jersey, USA, model C2282) equipped with a 720 nm cut-on filter. Cattle blood (0.020 mL) was collected from beef stew purchased from grocery stores. The approval for the use of cattle blood is granted by the Jackson State University Institutional Biosafety Committee (IBC). SO_3_SQ was synthesized as previously described.^40^ Bovine serum albumin (BSA) and human serum albumin (HSA) were purchased from Sigma-Aldrich (St Louis, MO).

### 2.2 Absorption and emission of SO_3_SQ

SO_3_SQ (0.0015 g) was dissolved in MilliQ H_2_O (1 mL) to make a dye stock solution. The stock solution (100 μL) was diluted in 1900 μL H_2_O (dilution factor 1 : 20) in one cuvette, as the purely aqueous SO_3_SQ sample. In another cuvette, the dye stock solution (100 μL), H_2_O (900 μL) and HSA (1000 μL) (10 mg mL^−1^) were added in sequence, as the SO_3_SQ-HSA sample. Absorption spectra were recorded over the 400–1000 nm wavelength region, and emission spectra were recorded over the 700–850 nm wavelength range.

### 2.3 Absorption and emission of SO_3_SQ with different components

Various components such as NaCl, CaCl_2_, FeSO_4_, glucose, sucrose, urea, cysteine, BSA, HSA, and GSH were dissolved in Milli-Q water at a concentration of 5 mg mL^−1^. Milli-Q water was used as a control. Fluorescence spectroscopy of each sample was taken with an excitation wavelength of 693 nm before and after the addition of SO_3_SQ, with a spectrum acquisition range from 700 nm to 850 nm. The ratio of the fluorescence emission intensity at 758 nm after adding SO_3_SQ (*I*) to that before the addition SO_3_SQ (*I*_0_) was then calculated.

### 2.4 Emission of SO_3_SQ with blood

Cattle blood was diluted with phosphate buffer solution to 5.0 μL mL^−1^. SO_3_SQ (0.15 mg) was dissolved in Milli-Q water (2 mL) to get a concentration of 0.075 mg mL^−1^. The mixture of cattle blood and SO_3_SQ was prepared by mixing blood (10 μL mL^−1^, 1 mL) with SO_3_SQ (0.15 mg mL^−1^, 1 mL). The fluorescence spectrum of the solution was collected at the excitation wavelength of 693 nm. In these studies, cattle blood was used as the blood source since SO_3_SQ has shown the ability to detect both human and bovine albumin.^43^

### 2.5 Stability of SO_3_SQ fluorescence emissions

The emission spectra of SO_3_SQ were record after 18 days in solutions containing salts (NaCl, CaCl_2_, and FeSO_4_), small molecules (glucose, sucrose, urea, cysteine, and glutathione), and proteins (BSA and HSA). The peak intensity ratios for fluorescence intensity at 758 nm from before and after this time frame are studied. All solutions were kept at 4 °C under dark.

### 2.6 Detection of bloodstains at different dilution factors

The cattle blood solution was diluted in phosphate-buffered saline with the factors of 1 : 1, 1 : 10, 1 : 100, 1 : 500, and 1 : 1000, respectively. The dilutions allow for the sensitivity of the SO_3_SQ dye to be shown, which is important for attempted blood removal situations. The solutions were then dropped on a plastic surface and allowed to dry in air. The SO_3_SQ solution with a concentration of 0.075 mg mL^−1^ was subsequently sprayed on the bloodstains, and photos were taken after 5 min under both ambient light and NIR light. The typical imaging conditions are listed in Table S1.† The possible light sources and cameras in addition to the ones already mentioned in the study are listed in Tables S2 and S3.†

### 2.7 Detection of latent bloodstains

The bloodstain was first prepared with cattle blood without dilution. It was then scrubbed with a cloth using water, resulting in and invisible latent bloodstain under ambient light. Afterward, the surface was sprayed with SO_3_SQ solution, and illuminated with NIR light for the photos acquired. Because SO_3_SQ is very sensitive, SO_3_SQ can be used for detection of bloodstains in a broad range of concentrations. The optimal concentration of SO_3_SQ could be close to the concentration of albumin to detect. It will work if the concentration of SO_3_SQ is ten times lower than the concentration of albumin.

### 2.8 Stability of dye with bloodstain

A mock crime scene was created by spattering blood solution with a diluted factor of 1 : 500 on a plastic surface. After a while, the bloodstain was sprayed with SO_3_SQ and kept in the air for 30 min, 2 hours, 3 days, and 7 days. We then observed the fluorescence emission and photographed under NIR illumination at each interval.

### 2.9 Detection of old latent bloodstains

A blood solution was dripped onto a plastic surface and then wiped off after waiting for ten minutes. The bloodstain was kept at room temperature for 1 day, 3 days, and 7 days. At every defined time, the bloodstain was sprayed with SO_3_SQ and illuminated under NIR light.

### 2.10 Blood fingerprint visualization

The thumb with diluted cattle blood (dilution factor 1 : 100) was pressed onto an aluminum soda can. Then SO_3_SQ solution was sprayed on the area with blood fingerprint and left to stand for 5 minutes. The photo was taken under NIR light.

### 2.11 Agarose gel electrophoresis

A stock solution of DNA (160 μM) in deionized water was incubated with deionized water or SO_3_SQ with different concentrations (0.1 mM, 0.5 mM, and 1.0 mM) for 24 h, respectively. The Agarose gel (1%) was prepared using agarose powder and dissolved in TBE (1 × Tris/boric acid/EDTA) buffer. After DNA gel loading dye (1 μL, 6×, Thermo Scientific™) was mixed with the sample solutions (9 μL), all samples were loaded into the wells of the gel. The gel was electrophoresed on the FisherBiotech Electrophoresis Systems Mini-Horizontal Unit FB-SB-710 for 60 min at 100 V. Then, the gel was stained with SYBR Safe DNA gel stain (Invitrogen by Thermo Fisher Scientific). Lastly, the gel was imaged by the Gel Doc XR documentation system.

## 3. Results and discussion

**Fig. 1:**
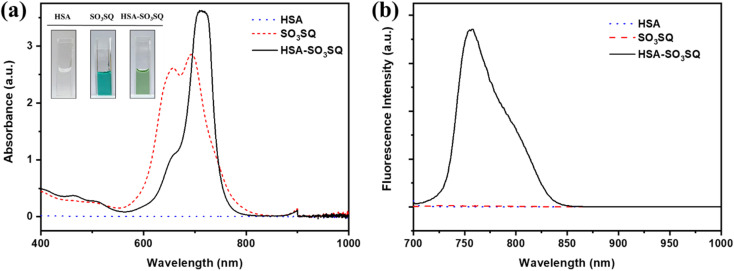
(a) Absorption and (b) emission spectra of pure HSA (5.0 mg mL^−1^), pure SO_3_SQ (0.075 mg mL^−1^) and the mixture of HSA with SO_3_SQ in aqueous solution. The excitation wavelength is 693 nm. Inset is the photograph for three solutions.

SO_3_SQ was synthesized as previously described^40^ and the molecular structure is shown in Scheme 1. First, the absorption and emission properties of the dye were studied. As seen in Fig. 1a, the aqueous solution of SO_3_SQ gives absorption at maximum at 690 nm and a prominent shoulder feature at 658 nm which is consistent with previous work.^40^ When human serum albumin (HSA) was added, the absorption wavelength of SO_3_SQ mixture red shifts to an absorption maximum of 712 nm. Meanwhile, the absorption spectrum of HSA shows no absorption from 400 nm to 1000 nm. A change in the solution color is also observed between the two solution conditions. In aqueous solution the higher energy absorption feature of SO_3_SQ results in a blue color (inset of Fig. 1a), while after the addition of HSA the color changes to green which is consistent with the red shift in the absorption maxima of the dye. SO_3_SQ shows minimal fluorescence when excited at 693 nm in aqueous solution, a useful feature for blood stain detection since this gives a low background signal (Fig. 1b). However, a strong emission feature is observed for a mixture of SO_3_SQ with HSA. This dramatic increase in emission intensity indicates a productive interaction between SO_3_SQ and HSA that promotes radiative decay and results in a turn-on feature that is useful in blood stain detection.

**Fig. 2:**
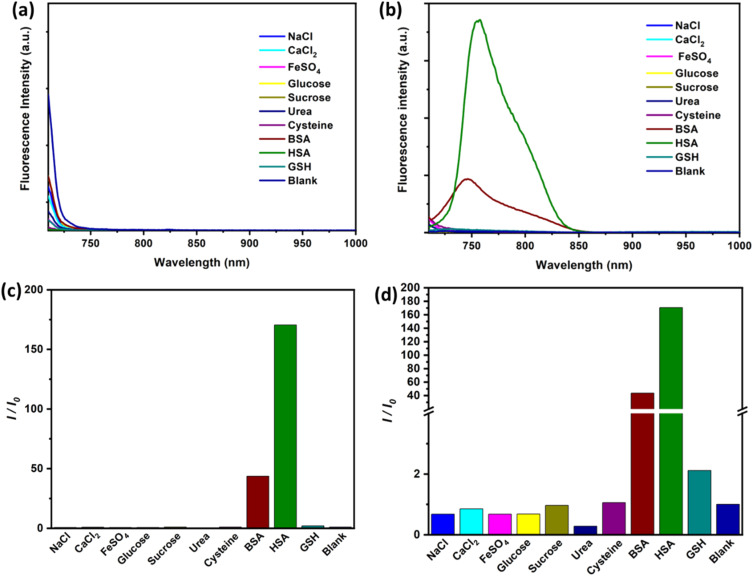
Fluorescent spectra of common components in bodily fluids (5.0 mg mL^−1^) before (a) and after (b) adding SO_3_SQ (0.075 mg mL^−1^) at excitation wavelength of 693 nm. (c) The on-off fluorescent ratio (*I*/*I*_0_) of fluorescence emission of SO_3_SQ with common components in bodily fluids at emission of 758 nm. (d) Enlarged on-off fluorescent ratio to show the small change on fluorescent intensity of other bodily fluids.

To further evaluate the specificity of SO_3_SQ interacting with HSA, the absorption and emission of the dye with common components in bodily fluids are studied. Fluorescence spectroscopy of the solution of the bodily fluid components was studied before and after the addition of SO_3_SQ. Without SO_3_SQ, all of the solutions show negligible emission when excited at 693 nm (Fig. 2a). Upon addition of the SO_3_SQ dye, strong emission was observed for bovine serum albumin (BSA) and HSA, and little to no fluorescence was observed for the salt solutions (NaCl, CaCl_2_, FeSO_4_) and small molecule solutions (glucose, sucrose, urea, cysteine, glutathione) (Fig. 2b). A quantitative comparison of the solutions shows the on-off intensity ratio (*I*/*I*_0_) increases about 160 times for the HSA solution and about 50 times for the BSA solution (Fig. 2c). The on-off intensity ratios for other bodily fluids are close to one (Fig. 2d). The high intensity ratio indicates that SO_3_SQ is selectively turned on in the presence of serum albumin, especially to human serum albumin. This validates the use of SO_3_SQ for bloodstain detection since serum albumin is present in high concentrations in the blood.^44^

**Fig. 3:**
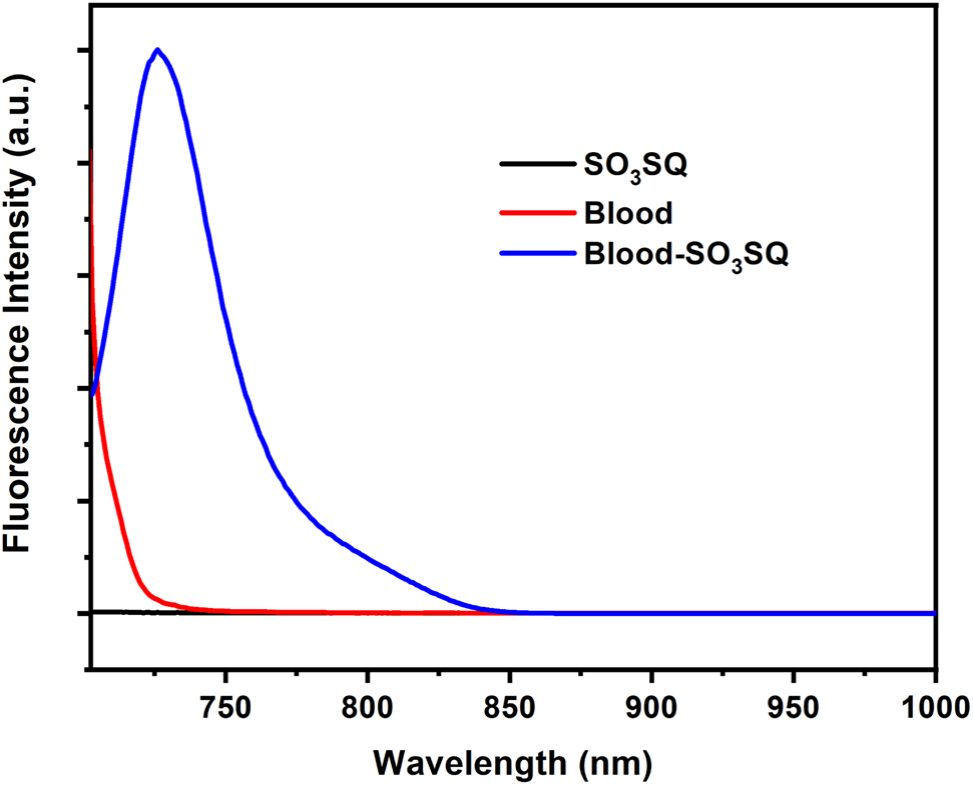
Fluorescence of SO_3_SQ (0.075 mg mL^−1^), cattle blood (5.0 μL mL^−1^), and blood-SO_3_SQ at 693 nm excitation wavelength.

Given the selectivity of SO_3_SQ for serum albumin detection, we evaluated the ability of the dye to demonstrate the same “turn-on” emission for blood solution. Cattle blood was selected as a model in this study for its easy access and the similar structures of BSA and HSA which both show turn-on behavior. As expected, there is a strong fluorescence enhancement in the blood immediately after being mixed with SO_3_SQ; however, SO_3_SQ and the pure blood sample separate display little to no fluorescence when excited at 693 nm (Fig. 3). This finding is consistent with the emission studies in the solution of HSA. At the excitation wavelength of 660 nm, the profiles of fluorescent emission spectra are similar except that intensity of fluorescent emission is relatively lower at 660 nm compared to 693 nm (Fig. S2†).

**Fig. 4:**
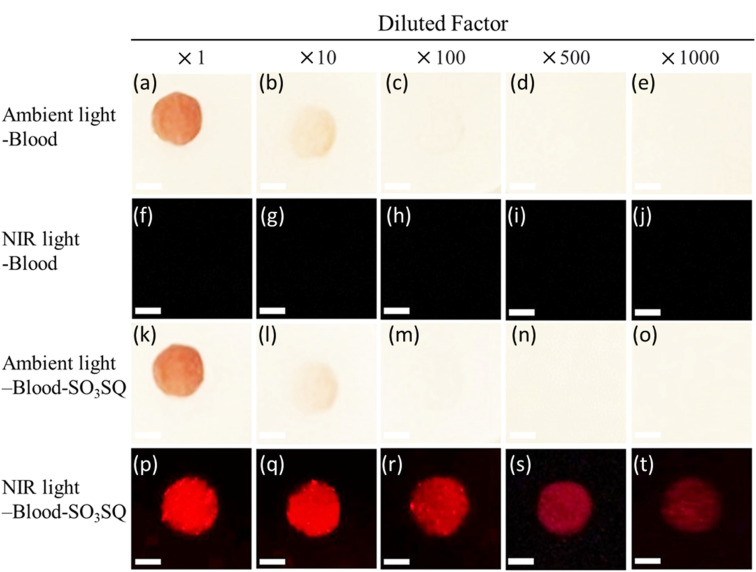
Bloodstain visualization with SO_3_SQ at different dilution factors. (a–e) Bloodstain photographs taken under ambient light. (f–j) Fluorescent photographs taken under NIR light (660 nm). (k–o) Photograph after spray of SO_3_SQ under ambient light. (p–t) Photograph after spray of SO_3_SQ under NIR light (660 nm). The concentration of SO_3_SQ is 0.075 mg mL^−1^. The scale bar: 5 mm.

The turn-on mechanism of SO_3_SQ illustrates its potential application in bloodstain visualization. To investigate the ability of the dye to detect dilute blood stains, blood solutions were diluted in phosphate-buffered saline in dilution factors of 1 : 1, 1 : 10, 1 : 100, 1 : 500, and 1 : 1000, respectively. Then, they were dropped on a plastic surface and dried in air. Under ambient light, the bloodstains cannot be identified by the human eye at concentrations at or below the 1 : 100 dilution (Fig. 4a–e). The bloodstains by themselves are also non-observable under NIR light (Fig. 4f–j). SO_3_SQ solution was subsequently sprayed on the bloodstains, and photos were taken under ambient light (Fig. 4k–o). Bloodstains sprayed with SO_3_SQ were visualized under illumination with a NIR lamp (660 nm) and visualization with an NIR camera for all dilutions studied herein (Fig. 4p–t). The ability to detect highly dilute blood stains demonstrates the high sensitivity of the SO_3_SQ dye for bloodstain detection.

**Fig. 5:**
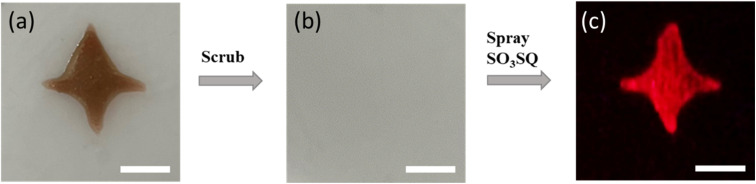
The visualization of bloodstain after washing. The photographs taken under ambient light (a) before and (b) after the bloodstain was scrubbed. (c) The photographs taken under NIR light after bloodstain was scrubbed and SO_3_SQ was sprayed. The concentration of SO_3_SQ is 0.075 mg mL^−1^. The excitation wavelength is 660 nm. The scale bar: 10 mm.

SO_3_SQ was further evaluated for the detection of latent bloodstains in a mock crime scene. A bloodstain on plastic surface was first prepared without dilution (Fig. 5a). The bloodstain was then scrubbed with a cloth using water, resulting in an invisible latent bloodstain under ambient light (Fig. 5b). Afterward, the surface was sprayed with SO_3_SQ solution. Under the NIR irradiation, SO_3_SQ enabled visualization of the latent bloodstain in high resolution (Fig. 5c). These promising results indicate that such technology can be used to visualize latent bloodstain patterns under NIR light, and open a new path for the development of forensic technology in crime scene investigation and reconstruction.

**Fig. 6:**
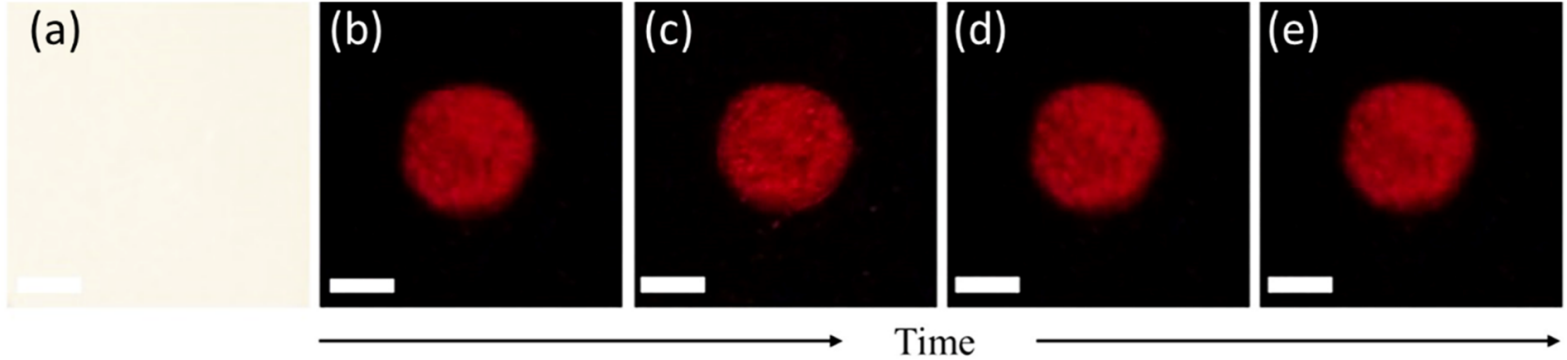
The bloodstain visualization for an extended time. (a) Typical latent bloodstains in ambient light. Photographs taken under NIR after (b) 0.5 h, (c) 2 h, (d) 3 days, and (e) 7 days after spraying with SO_3_SQ. The excitation wavelength is 660 nm. Scale bar: 5 mm.

Compared to the existing method of luminol chemiluminescence (CL) which has a very short duration, the fluorescence emission of SO_3_SQ is stable for several days post-application. The fluorescence spectra of different components mixed with the SO_3_SQ after 18 days are shown in Fig. S1a† and the peak intensity ratios are summarized in Fig. S1b.† Negligible changes in the emission intensity ratios were observed over the time period. As shown in Fig. 6, a mock crime scene is created by spattering blood solution. Then, the bloodstain was sprayed with SO_3_SQ and kept in the air over time. Photos were taken under NIR irradiation after 30 min, 2 hours, 3 days, and 7 days, respectively. The fluorescence pattern maintained a very strong intensity and demonstrated no significant decrease even after 7 days. In this way, SO_3_SQ demonstrates excellent chemical, photo, and oxidative stability under ambient conditions during the 7 days period. To better illustrate the capability of detecting aged bloodstains, we applied dye to latent bloodstains that had been aged for 1 day, 3 days, and 7 days. Subsequently, we observed the fluorescence emission at each time point. The results revealed distinct fluorescent patterns on all aged bloodstains, including those aged for 7 days (Fig. S3†). Therefore, this test underscores the sensitivity of SO_3_SQ in detecting bloodstains up to one week after the occurrence of the crime.

**Fig. 7:**
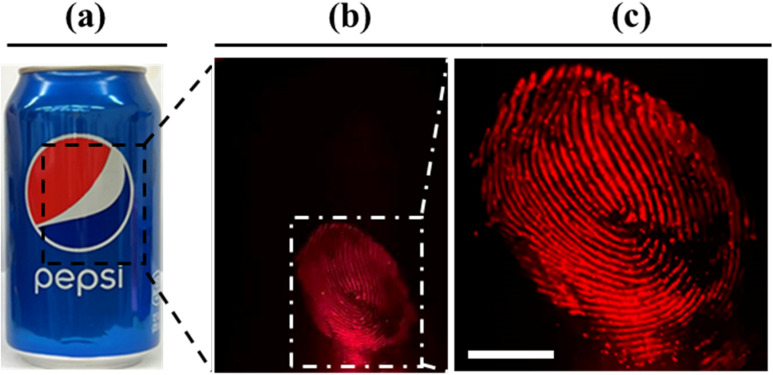
The visualization of a latent blood fingerprint. Photos of a latent blood fingerprint developed with blood taken under (a) ambient light, (b) NIR (660 nm) light irradiation, (c) enlarged three times for the selected area. Scale bar: 4.5 mm.

Fingerprints can provide crucial evidence in forensic investigations and identification of suspects. The visualization of fingerprints *via* bloodstains would prove to be of paramount importance in forensic evaluations. Herein, the detection of fingerprints *via* a bloodstain pattern from a complex background was evaluated. The fingerprints were prepared by a right thumb dipped in diluted blood and were deposited on the surface of an aluminum can. The latent fingerprint itself was not visible under ambient light (Fig. 7a); however, after treatment with SO_3_SQ, the fingerprint can be observed in high resolution under NIR light irradiation and visualized with a NIR camera (Fig. 7b). The enlarged area of a small part of the fingerprint is shown in Fig. 7c. It is important to note that a high-resolution fingerprint with clear end points and intersections is invaluable and visualizable *via* this technique. Therefore, this technique is not only useful for bloodstain detection, but for the resolution of fine details like those found in fingerprints.

Furthermore, we demonstrate that SO_3_SQ would not degrade DNA when interacting with blood. After incubating with SO_3_SQ at different concentrations for 24 hours, the structure change of DNA was evaluated by the gel electrophoresis. As shown in Fig. S4,† SO_3_SQ does not break down DNA or interface the structure of the DNA even in high concentration (up to 1 mM). The non-destructive property is very useful because blood samples are often collected for DNA test after blood is located during a criminal investigation.

## 4. Conclusions

In conclusion, this study has demonstrated a new mechanism for the detection of latent bloodstains based on the interaction of SO_3_SQ, a NIR fluorescent dye, with blood. Although many options are available to detect and visualize latent bloodstains, detection with greater selectivity and sensitivity is still desirable. The fluorescent dye, SO_3_SQ, exhibits very weak fluorescence on its own but gives strong emission upon mixing with HSA. This quantitative and qualitative study demonstrates that the emission intensity can increase roughly 160 times in the presence of HSA whereas negligible emission is observed for other common components in bodily fluids. Consistent with specific interaction of SO_3_SQ with HSA and BSA, the dye can be turned on when mixed with real blood samples. This turn-on fluorescent property of SO_3_SQ illustrates its potential for highly sensitive bloodstain visualization even after a blood sample is diluted 1000 times. In the mock crime scene, SO_3_SQ can visualize a latent bloodstain even after washing the bloodstain away to the point that it is invisible to the eye under ambient lighting. Further, the bloodstains sensitized with SO_3_SQ can be visualized for up to 7 days. This method is also shown to detect blood fingerprints with minimal background interference. The fingerprint patterns were observed to maintain high-resolution fingerprint lines with clear end points and intersections. Due to the non-destructive nature, the method would not destroy the DNA in blood as well. Overall, this study represents a new method for latent blood detection with high specificity and simple detection procedure through NIR illumination which reduces background auto-fluorescence. This study provides a novel principle for developing new technologies for latent blood detection and beyond.

## Author contributions

Conceptualization, data curation, visualization, writing – original draft, Jing Qu; methodology, data curation, validation, William Meador; data curation, validation, Pohlee Cheah; writing – review & editing, Eden E. L. Tanner; writing – review & editing, methodology, Jared Delcamp; conceptualization, writing – review & editing, funding acquisition, project administration, Yongfeng Zhao.

## Conflicts of interest

The authors declare no conflict of interest.

## Supplementary Material

RA-013-D3RA04320G-s001
