# Supplementary material for: Latent bloodstain detection using a selective turn-on NIR fluorescence dye responsive to serum albumin
Source: RSC Adv. 2023 Sep 15;13(39):27549–57. doi: 10.1039/d3ra04320g (PMC10502805; doi:10.1039/d3ra04320g)
Supplement: RA-013-D3RA04320G-s001 [file RA-013-D3RA04320G-s001.pdf]

Electronic Supplementary Material (ESI) for RSC Advances.
This journal is © The Royal Society of Chemistry 2023

Supporting information

Latent bloodstain detection using a selective turn-on NIR fluorescence dye responsive to serum albumin

Jing Qu,[a] *William Meador,*[b] *Pohlee Cheah,*[a] *Eden E. L. Tanner,*[b] *Jared Delcamp,*[b,*] *and Yongfeng Zhao*[a,*]

[a] Department of Chemistry, Physics & Atmospheric Sciences, Jackson State University, Jackson, MS 39217, USA

[b] Department of Chemistry and Biochemistry, University of Mississippi, University, MS, 38677, USA

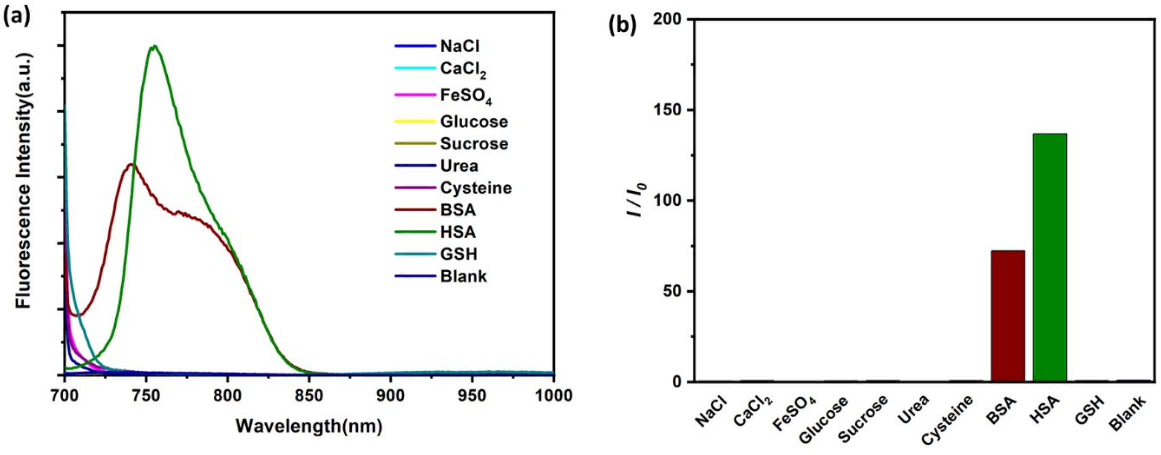

Figure S1. Fluorescence of body fluids with SO_3SQ after 18 days at 693 nm excitation wavelength.

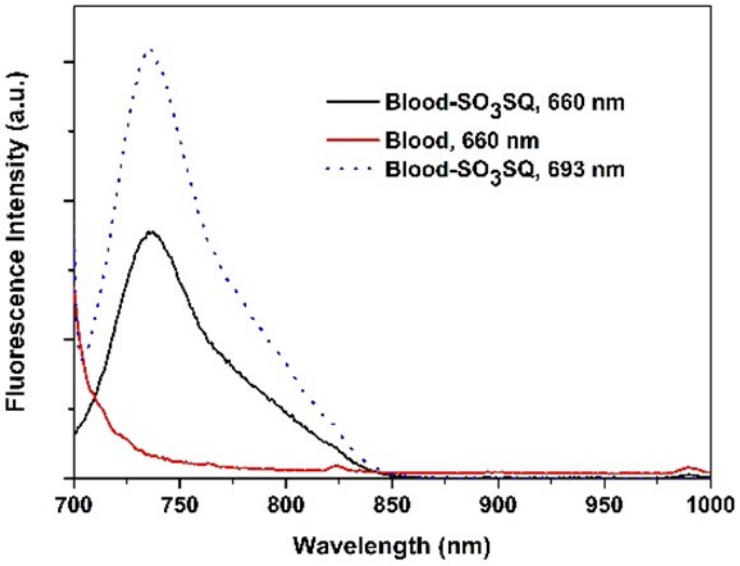

Figure S2. Fluorescent intensity of cattle blood (5.0 μL/mL) and blood-SO_3SQ at excitation wavelength of 660 nm and 693 nm.

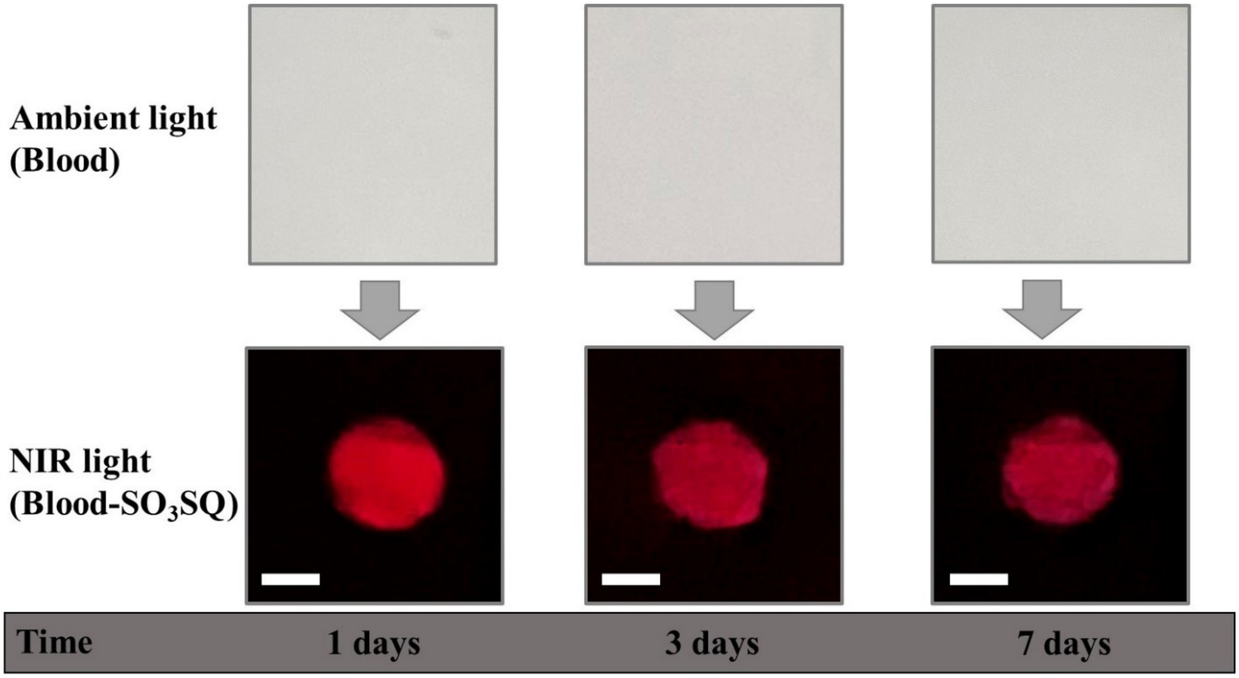

Figure S3. The visualization of aged bloodstain. The photographs were taken under ambient light and under NIR light after bloodstain was aged for 1, 3, and 7 days, respectively. The concentration of SO_3SQ is 0.075 mg/mL. The scale bar: 8 mm.

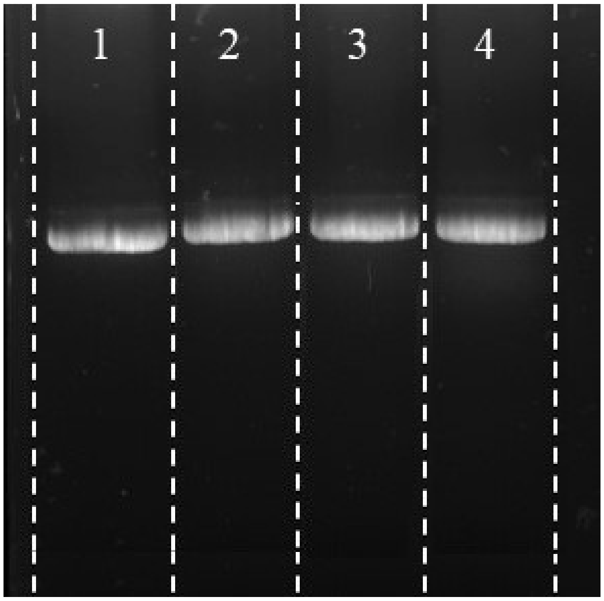

Figure S4. Agarose gel electrophoresis of DNA. Lane 1: no treatment (control); Lane 2: treated with 0.1 mM SO_3SQ; Lane 3: treated with 0.5 mM SO_3SQ; Lane 4: treated with 1.0 mM SO_3SQ.

Table S1. The details of the illuminating and detecting optics

The exciting light flux	26.8 mW/cm^2
The numerical aperture used for excitation	20 cm
Focal length of the lens used for excitation	300 mm
The resolution of the camera (pixel-size)	20 million
The exposure type	manual /automatic
The working distance of the camera for the optimal imaging	70 cm

Table S2. The list for applicable light sources

Item	Brand	model
Predator UV/Red/Green Light Blacklight Flashlight Blood Tracking Hunting Torch	VASTFIRE	LTB96
Nicron B70 LED Twist Rechargeable Flashlight	NICRON	B70
Cosmoing 400 Yards Tactical Red Gun Light Rechargeable LED Flashlight 18650	Cosmoing	FL15-RD
Hunting Red Green Gun Light USB Rechargeable LED Tactical Flashlight Torch 18650	Bruggex	1102
Mini Powerful Red Light LED Flashlight Zoomable 3Modes Torch Night Vision Lamp	AMSUER	Red Light

Table S3. The list for applicable cameras

Item	Producing countries	Serial Number
Sony ZV-E10 Camera	Digital SLR	Sony ZV-E10 Mirrorless Camera with 16-50mm Lens
Canon EOS 2000D Rebel T7 DSLR Camera	Digital SLR	Canon EOS 2000D
NBD Digital Camera	Compact	ND
KODAK PIXPRO AZ421 Red Digital Camera	Digital SLR	AZ421
Nikon COOLPIX P1000 16MP Digital Camera	Point & Shoot	Nikon Coolpix P1000
